# Juanbi Lijieqing Decoction Inhibits TLR4/NF-κB Signaling Pathway by Promoting PPARγ Expression to Relieve Acute Gouty Arthritis

**DOI:** 10.2174/0113862073378606250616114958

**Published:** 2025-07-03

**Authors:** Chengyin Lu, Fangxiao Zhu, Zhiqiang Luo, Hui Xiong, Yuxing Guo

**Affiliations:** 1 The Second Clinic College of Traditional Chinese Medicine, Hunan University of Chinese Medicine, Changsha, China;; 2 Department of Orthopedics, The First Hospital of Hunan University Chinese Medicine, Changsha, China;; 3 Department of Orthopedics, The Affiliated Hospital of Hunan Academy of Traditional Chinese Medicine, Changsha, China

**Keywords:** Juanbi Lijieqing Decoction, TLR4, NF-κB, PPARγ, acute gouty arthritis, chinese medicine, uric acid

## Abstract

**Introduction:**

This study aimed to investigate the mechanism of Juanbi Lijieqing Decoction (JLD) in alleviating acute gouty arthritis (AGA) by modulating PPARγ expression to suppress the TLR4/NF-κB pathway.

**Methods:**

A total of 84 male SD rats were divided into 7 groups of 12 rats. One group was randomly selected as the normal control group (Group A), while the remaining 72 rats were used to establish an acute gouty arthritis model through intraperitoneal injection of potassium oxonate combined with MSU ankle joint injection. These rats were randomly assigned to the model group (Group B), the high-dose Juanbi Lijieqing Decoction group (Group C), the medium-dose group (Group D), the low-dose group (Group E), the etoricoxib group (Group F), and the pioglitazone group (Group G), with 12 rats per group. The acute gouty arthritis model was established by intraperitoneal injection of potassium oxonate, followed by monosodium urate (MSU) injection into the ankle joint, and then by pharmacological intervention in each group. The ankle swelling index, pain threshold changes, and serum uric acid levels were observed in each group of rats. The pathological state of synovial tissue in each group was evaluated by hematoxylin-eosin (HE) staining. The levels of TNF-α, IL-6, and IL-1β were detected by enzyme-linked immunosorbent assay (ELISA). The protein expressions of TLR4, NF-κB, and PPARγ were detected *in vivo* and *in vitro* using Western blot.

**Results:**

JLD effectively reduced local swelling, relieved pain, and lowered serum uric acid levels in rats with AGA. Both *in vivo* and *in vitro* experiments demonstrated that the Chinese medicine groups showed a significant reduction in TNF-α, IL-1β, and IL-6 levels. Moreover, in *in vivo* experiments, the expression of PPARγ protein was significantly upregulated in the JLD and pioglitazone groups, whereas the expressions of TLR4 and NF-κB p65 proteins were significantly downregulated, a pattern not observed in the etoricoxib group. *In vitro* experiments demonstrated significant increases in PPARγ protein expression in the pioglitazone and medicated serum groups, accompanied by significant decreases in TLR4 protein expression. Meanwhile, the NF-κB inhibitor group only exhibited a downregulation of TLR4 protein expression.

**Discussion:**

Our findings demonstrated that JLD alleviated acute gouty arthritis by upregulating PPARγ expression, which subsequently inhibited the TLR4/NF-κB signaling pathway. This mechanism effectively reduced inflammatory cytokine production (TNF-α, IL-1β, and IL-6), explaining the observed anti-swelling and analgesic effects.

**Conclusion:**

JLD mitigates AGA symptoms by promoting PPARγ, which in turn inhibits TLR4/NF-κB signaling, thereby reducing inflammation, uric acid, and joint swelling. This highlights the therapeutic potential of JLD for gout management, though long-term effects and molecular targets warrant further study.

## 1 INTRODUCTION

Gout, a chronic condition caused by a purine metabolic disorder and elevated blood uric acid levels, is characterized by repeated bouts of acute gouty arthritis [1]. As the disease progresses, it frequently impacts the kidneys, leading to gouty nephropathy and the formation of renal urate stones [2, 3]. Additionally, this condition also increases the risk of developing cardiovascular diseases [4]. In recent years, with the improvement of people's living standards, the change in dietary structure, sugar, fat, and protein intake has increased significantly. As a result, not only the prevalence of gouty arthritis has shown a trend of increasing year by year, but also the age of onset has been decreasing gradually, leading to both economic and social burdens [5-7]. Modern medicine often uses colchicine and non-steroidal anti-inflammatory drugs (NSAIDs) to control acute flares of gouty arthritis, but their side effects are substantial, including frequent adverse reactions, such as liver and kidney damage, gastrointestinal reactions, respiratory depression, and even bone marrow suppression, making the treatment of gout more challenging [8-10]. In contrast, Chinese medicine has an early understanding of gout, and its effectiveness and safety can be seen in long-term clinical applications. Therefore, the search for new anti-gouty arthritis drugs from traditional Chinese medicine will become a development direction and research hotspot in the future [11-19].

Juanbi Lijieqing Decoction (JLD) is a pure Chinese medicine compound created based on the therapeutic ideas of clearing heat, dispelling dampness, and dispersing blood stasis [20]. Our previous studies reported that JLD can significantly reduce blood uric acid levels, relieve joint symptoms, reduce inflammatory cell infiltration of synovial tissue, and has obvious anti-inflammatory and swelling effects, but its mechanism of action needs to be further elucidated.

Recent studies have shown that the acute flares of gouty arthritis are caused by the exfoliation of sodium urate crystals into the joint cavity, acting on macrophages, neutrophils, synovial cells, and monocytes, releasing a variety of inflammatory mediators, such as histamine, prostaglandins, IL-1β, IL-6, and TNF-α, causing an inflammatory cascade reaction [21-23]. The mediation of the TLR4/NF-κB signaling pathway plays an important role in this process [24, 25]. PPARγ can competitively inhibit the expression of the TLR4/NF-κB pathway and hinder the inflammatory response [26, 27]. Therefore, this study aims to observe the regulatory effect of JLD on TLR4/NF-κB through *in vitro* and *in vivo* experiments and explore its mechanism of action in the treatment of acute gouty arthritis.

## 2 MATERIALS AND METHODS

### 2.1 Experimental Animals, Cells, and Materials

All rats in this study were provided by the Animal Experiment Center of Hunan University of Chinese Medicine (Animal Certificate No.: SCXK (Xiang) 2021-0002). The rats were 6-8 weeks old male SD rats, with an average weight of 200 - 220 grams.

This study strictly adhered to the ARRIVE guidelines for reporting *in vivo* experiments. All procedures involving live animals were conducted in accordance with the highest ethical standards and the 3Rs principle (Replacement, Reduction, and Refinement). The study was approved by the Animal Ethics Committee of Hunan University of Chinese Medicine on April 18^th^, 2024 (Approval No.: HNUCM21-2404-202) and lasted for one month. The humane endpoint in this study was defined as a sustained weight loss exceeding 15% over one week, accompanied by loss of appetite or severe pain, difficulty in breathing, *etc*. This criterion was based on its ability to reflect the health and suffering status of the animals, determined according to relevant studies and guidelines. Professional staff closely monitored the animals, with timely assessment and intervention upon reaching the endpoint. Euthanasia was carried out by the exsanguination method. Before exsanguination, the animals were anesthetized by isoflurane inhalation. The rats were placed in a specialized anesthesia induction chamber, and the isoflurane concentration was adjusted to 5% (induction concentration) with an oxygen flow rate of 1-2L/min to rapidly induce anesthesia in the rats. Subsequently, the operator precisely severed the abdominal aorta stump and other major blood vessels, such as the inferior vena cava, using sterile surgical instruments to allow rapid blood loss. This procedure was performed swiftly and steadily to minimize the stress. Death was determined by observing that breathing stopped for at least 5 minutes, the heartbeat disappeared for at least 10 minutes, the corneal reflex disappeared, and the muscles were relaxed. The procedure was performed by trained professionals following sterile techniques and animal welfare principles. J774.1 Mouse mononuclear macrophage cells (batch number: CBR130812) were purchased from Saiqi Bioengineering Co., Ltd. (Shanghai, China). The sources of the reagents used in this study are provided in Table **1**.

### 2.2 Preparation of the Solution

#### 2.2.1 Preparation of *In Vivo* Experimental Solutions

##### 2.2.1.1 Preparation of Potassium Oxonate Solution

A total of 3g of potassium oxonate crystals were weighed and dissolved in 97 mL of normal saline to prepare 3% potassium oxonate solution.

##### 2.2.1.2 Preparation of Sodium Urate Solution

A total of 1250 mg of microcrystalline sodium urate was weighed, 45 mL of normal saline was added, and then 5 mL of polysorbate-80 was added, heated, and stirred to make a sodium urate solution with a concentration of 25 mg/mL.

##### 2.2.1.3 Preparation of JLD Solution

JLD (Table **2**) is a combination of 10 kinds of traditional Chinese medicine. All Chinese medicines were decocted and concentrated to the original drug concentration of 2.2g/mL by the Pharmacy Department of the Second Affiliated Hospital of Hunan University of Chinese Medicine, and the required concentration was prepared by adding distilled water before use. We have previously conducted a study on the identification of its components. The main active ingredients include baicalin, β-sitosterol, berberine, stigmasterol, quercetin, campesterol, naringin, kaempferol, and others.

##### 2.2.1.4 Preparation of Etoricoxib Solution

Etoricoxib tablets were finely powdered and dissolved in water to prepare a suspension containing a concentration of 1.1 mg/mL.

##### 2.2.1.5 Preparation of Pioglitazone Solution

Pioglitazone hydrochloride was combined with 1% CMC-Na to prepare a suspension containing 100 mg/50mL. The suspension was stored in the refrigerator at 4°C.

#### 2.2.2 Preparation of *In Vitro* Experimental Solutions

##### 2.2.2.1 Preparation of Sodium Urate Solution

A total of 5 mg of microcrystalline sodium urate was weighed, 45 mL of normal saline was added, and then 5 mL of polysorbate-80 was added, heated, and stirred to make a sodium urate solution with a concentration of 100 μg/mL.

##### 2.2.2.2 Preparation of NF-κB Inhibitor (SN50)

Under strict sterile conditions, 20 mg of SN50 was dissolved in distilled water, made into a concentration of 20 μmoL/L suspension, and stored at -20°C.

##### 2.2.2.3 Preparation of Pioglitazone Solution

Pioglitazone hydrochloride was added with 1% CMC-Na to prepare a suspension of 20 μmoL/L. The suspension was stored in the refrigerator at 4°C.

##### 2.2.2.4 Preparation of Medicated Serum

Twenty male SD rats (200±20 g, clean grade; provided by the University Animal Experiment Center) were randomly divided into the drug-containing serum group and the blank control serum group, with 10 animals in each group, according to the routine method of serum pharmacology, namely 3d(day)-2t(time)-1h(hour) mode. The drug-contain-ing serum group was intragastrically administrated with JLD at a dose of 44g/kg, while the blank control group was intragastrically administrated with an equal dose of normal saline twice a day for 3 days. One hour after the last administration, blood was collected from the abdominal aorta to collect the serum of the two groups of rats. Firstly, the animals were anesthetized by inhalation of isoflurane. Under the anesthetized state, the abdominal cavity of the rat was opened, and 4-6 ml of blood was collected using a blood collection tube. After centrifugation, the supernatant was taken and filtered through a 0.22 μm microporous filter membrane for sterilization and then stored in a freezer at - 20 °C.

### 2.3 Model Establishment of Acute Gouty Arthritis for *In Vivo* Experiments

For the establishment of the animal model, potassium oxonate was administered intraperitoneally at a dose of 1 mL/100g twice daily for 7 days to induce hyperuricemia. On day 8, 0.2ml of monosodium urate (MSU) (25 mg/mL) was injected into the right ankle joint cavity of the rats to induce acute gouty arthritis, and the blank group was injected with an equal dose of normal saline. The timing and dosage of these injections were selected based on the literature and previous studies from our research group, indicating that this protocol optimally induces hyperuricemia and the typical acute inflammatory response of gout, ensuring the consistency and reliability of the model [20, 28].

### 2.4 Cell Culture *In Vitro* Experiments

The J774.1 mononuclear macrophage strain was cultured in a flask (containing 10% fetal bovine serum, 100 U/mL penicillin, and 100 μg/mL streptomycin) in a 37°C, 5% CO_2_, 100% relative humidity incubator. When the cells reached approximately 80–90% confluence, they were passaged at a 1:3 ratio [29].

### 2.5 Grouping and Intervention

#### 2.5.1 Grouping and Intervention in *In Vivo* Experiments

After feeding 84 male SD rats for 1 week, 12 rats were randomly selected as a blank group, namely the normal control group (Group A), and the remaining 72 were modeled. After modeling, they were randomly divided into 6 groups, with 12 animals in each group, namely the model group (Group B), JLD high-dose group (Group C), JLD medium-dose group (Group D), JLD low-dose group (Group E), etoricoxib group (Group F), and pioglitazone group (Group G). The sample size of 12 rats per group was determined using statistical power analysis to achieve 80% power at a 0.05 significance level, assuming a medium effect size. This sample size was selected based on similar studies of acute gouty arthritis, ensuring reliable and statistically significant results while minimizing the risk of type II errors. The pharmacological intervention was given immediately after intra-articular injection. According to the equivalent dose ratio table based on body surface area converted between humans and animals, rats in each JLD low-dose group were given 11g/kg of JLD daily. According to the ratio of low, medium, and high dosages of 1:2:4, the daily doses of medium and high dose groups were 22 g/kg and 44 g/kg, respectively. The daily dose of the etoricoxib group was 11 mg/kg, and the daily dose of the pioglitazone group was 20 mg/kg [30]. Rats in normal control and model groups were gavaged with normal saline of 20ml/kg. Each rat was administered 2 times daily for 2 days continuously.

#### 2.5.2 Grouping and Intervention in *In Vitro* Experiments

The cells were passaged to a 6-well plate, and after 24 hours, they were randomly divided into 6 groups, namely normal control group (Group A), model group (Group B), pioglitazone group (Group C), NF-κB blocker group (Group D), blank serum group (Group E), and drug-containing serum group (Group F), and 5 complex wells were set up in each group. No other components were added to the normal control group culture medium, and the final concentration of 100 μg/mL sodium urate was added to the remaining group culture medium [31]. Then, 20 μmol/L of pioglitazone was added to the culture medium of the pioglitazone group, and 20 μmol/l of NF-κB blocker (SN50) was added to the culture of the NF-κB blocker group. Furthermore, 10% of rat blank serum was added to the blank serogroup culture medium. In the drug-containing serum group, 10% of rat drug-containing serum was added, and the culture continued for 48 hours.

### 2.6 Measurement of Swelling Index

The joint circumference at 4 hours, 12 hours, 24 hours, and 48 hours before and after molding was measured using a 2-3 mm wide paper strip and a four-use stainless steel band gauge caliper. The joint swelling index was calculated as:

Joint swelling index = circumference after molding - circumference before molding/circumference before molding.

In order to ensure the accuracy of the measurement as much as possible, the entire experiment was performed by the same person, and each group was averaged three times repeated.

### 2.7 Pain Threshold Measurement

Using the tail flicking method and a constant temperature water bath at 55°C as the source of thermal pain, the tip of the tail was put 5 cm above hot water, and the time was recorded from water ingress to shrinkage of the tail before administration. After 48h post-administration, it was measured twice (with an interval of 10 min), and its average value was calculated.

### 2.8 Blood Uric Acid Determination

The yellowish upper serum was collected and detected using a fully automatic biochemical analyzer.

### 2.9 Observation of Histopathological Changes in Ankle Synovium

Hematoxylin-eosin (HE) staining was used to observe synovial tissue morphology.

### 2.10 Determination of TNF-α, IL-1β, and IL-6 Contents

The contents of TNF-α, IL-1β, and IL-6 in the synovial tissue of experimental rats and the supernatant of experimental cells *in vitro* were determined by enzyme-related immunosorbent assay (ELISA) in strict accordance with the instructions.

### 2.11 Determination of PPARγ, TLR4, and NF-κB Proteins

Western blotting (WB) was used to detect the protein expression levels of PPARγ, TLR4, and NF-κB in synovial tissues of rats from *in vivo* experiments and in cells from *in vitro* experiments.

### 2.12 Data Analysis

Data were analyzed using SPSS 19.0 software and are expressed as mean ± standard deviation (SD). For comparisons among multiple groups, one-way analysis of variance (ANOVA) was used, followed by Tukey’s post-hoc test for pairwise comparisons when a significant difference was found. If variances were unequal, Dunnett’s T3 test was conducted. For repeated measures data, such as the ankle swelling index measured over time, repeated measures ANOVA was used. Non-normally distributed histopathological data were analyzed using the Mann-Whitney U test and Kruskal-Wallis test for comparisons across multiple groups. Cytokine levels and protein expression data were also analyzed by one-way ANOVA, with Tukey’s post-hoc test or Bonferroni correction for multiple comparisons. T-test was applied for comparisons between two groups when appropriate. *P<*0.05 was considered to be statistically significant.

## 3 RESULTS

### 3.1 General Condition of the Animals

The general condition of all animals was observed and recorded throughout the experiment. During the adaptation phase before modeling, rats in all groups were active, with normal food and water intake, smooth and shiny fur, and no abnormal behaviors or signs. After administration of 10% chloral hydrate, no peritoneal inflammation symptoms were observed in any of the animals. Their abdomens were soft without tenderness, and no fever, vomiting, or diarrhea occurred. After modeling, rats in the model group and each drug intervention group gradually exhibited typical symptoms of acute gouty arthritis, such as redness and swelling of the ankle joint, limited movement, *etc*., following injection of oxypurinol and sodium urate solution. However, their mental state remained acceptable, and while food intake was slightly reduced compared to pre-modeling, they were still able to eat and drink independently. The weight of the animals at the time of sacrifice ranged from 260g to 300g, and the exsanguination volume of blood was within the range of 18-20 ml.

### 3.2 JLD has the Effect of Reducing Swelling, Relieving Pain, and Lowering Uric Acid

**Fig. (1):**
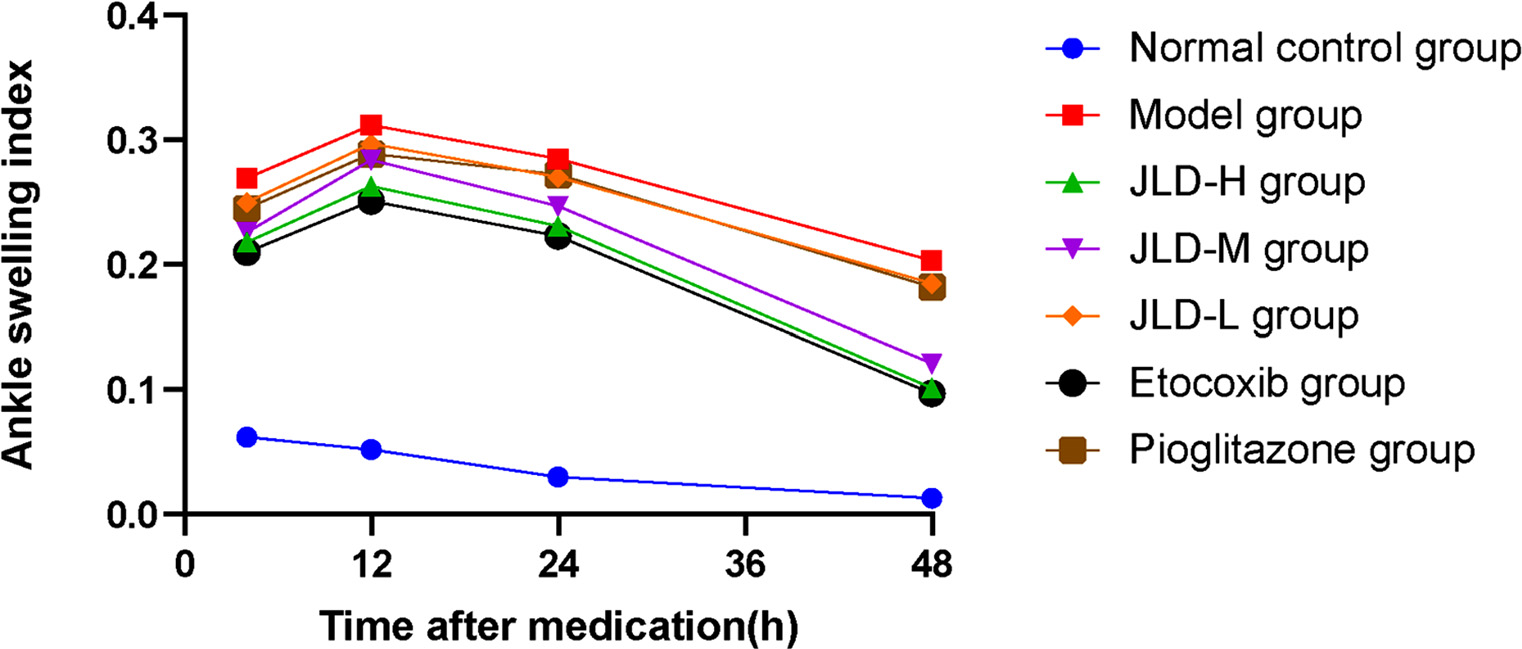
Ankle swelling index plot.

**Fig. (2):**
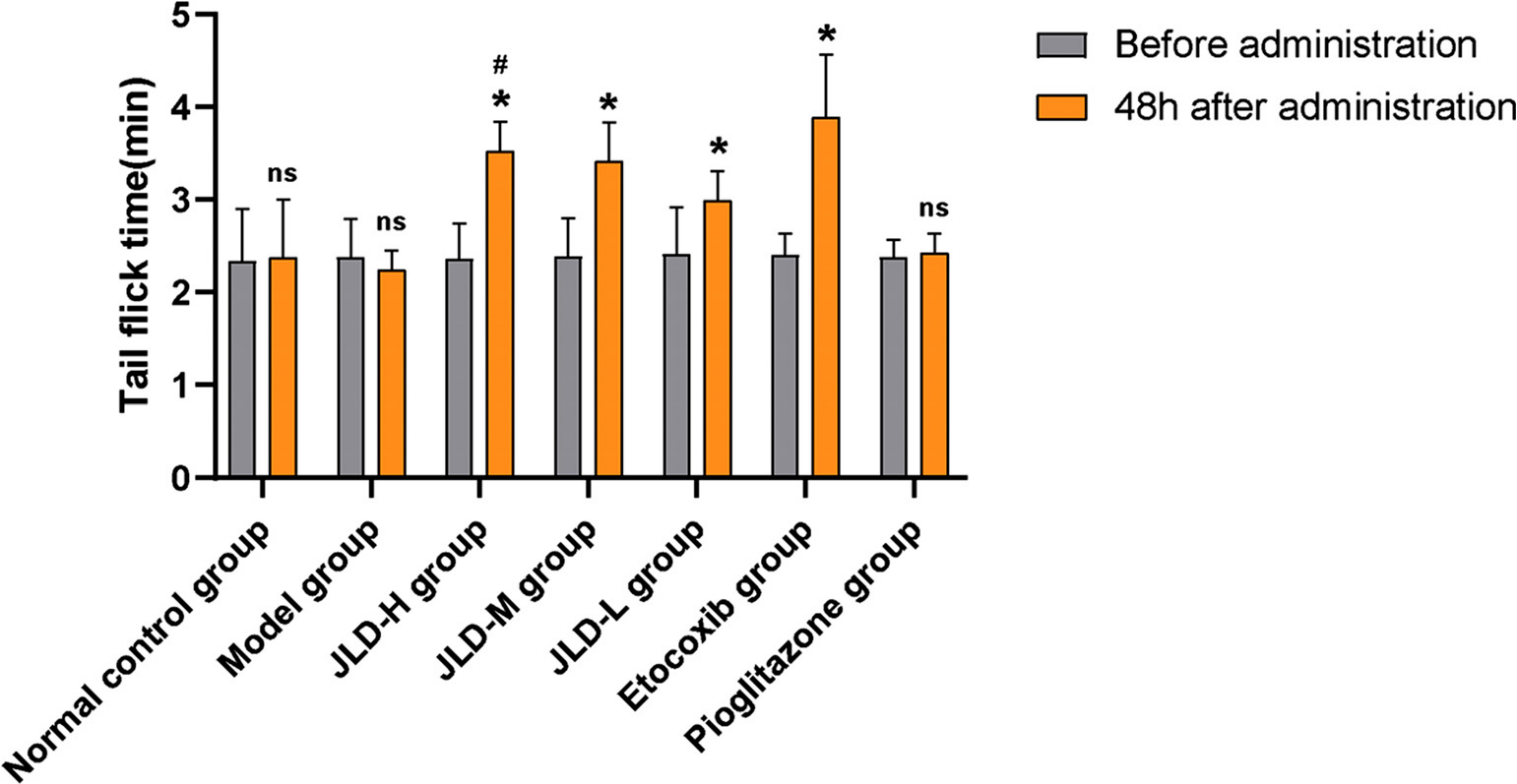
Pain threshold change chart: (Compared with the group before medication, *P<0.05; after 48 hours of medication, compared with the low-dose JLD group, *^#^P<0.05*; compared with the model group with no significant difference, ^ns^*P>0.05*).

**Fig. (3):**
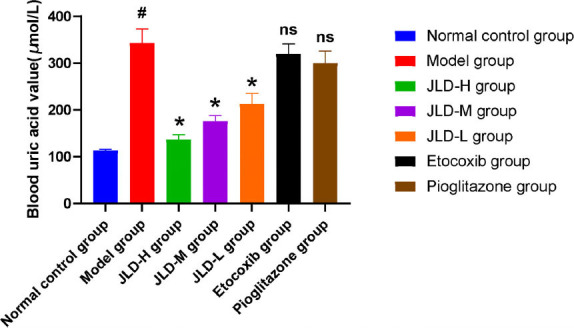
Blood uric acid level in each group: (Compared with the normal control group, *^#^P<0.01*; compared with the model group, *P<0.05; compared with the model group with no significant difference, ^ns^*P>0.05*).

In *in vivo* experiments, we measured ankle swelling index, pain threshold changes, and blood uric acid levels in each group of rats. The swelling index results (Fig. **1**) showed that compared with the model group, the swelling index of each JLD dose groups, etoricoxib group, and pioglitazone group was reduced to varying degrees, and the swelling index of the JLD-H group was smaller than that of the JLD-M group and the JLD-L group. It was suggested that the JLD reduced swelling of local tissues of gouty arthritis in rats, and the higher the dose, the stronger the effect. The results of pain threshold change (Fig. **2**) showed that the reaction time of tail flicking in rats was significantly prolonged in each JLD dose group and etoricoxib group after treatment (*P<0.05*), while there was no significant change in the normal control group, model group, and pioglitazone group (*P>0.05*). Before administration, there was no significant difference in the tail-flicking reaction time between groups (*P>0.05*). After administration, the tail-flicking reaction time of rats in the JLD-H group was significantly prolonged compared with the JLD-L group *(P<0.05)*. It showed that the JLD had an analgesic effect, and the high dose effect was the most effective. The results of blood uric acid levels in each group (Fig. **3**) showed that the blood uric acid values of rats in the model group were significantly higher than those in the normal control group (*P<0.05*), indicating that the continuous intraperitoneal injection of potassium oxonate was successful in modeling hyperuricemia. After treatment, the blood uric acid levels in the low, medium, and high-dose JLD group decreased significantly compared to the model group *(P<0.05)*, indicating a clear uric acid-lowering effect of JLD that progressively strengthened with increasing doses.

### 3.3 JLD can Improve Local Pathological Changes

In *in vivo* experiments, when dissecting the ankle joint, it was found that the tissue structure of the normal control group was normal and clear, while in the model group and various drug groups, urate crystallization was found in the ankle cavity.

**Fig. (4):**
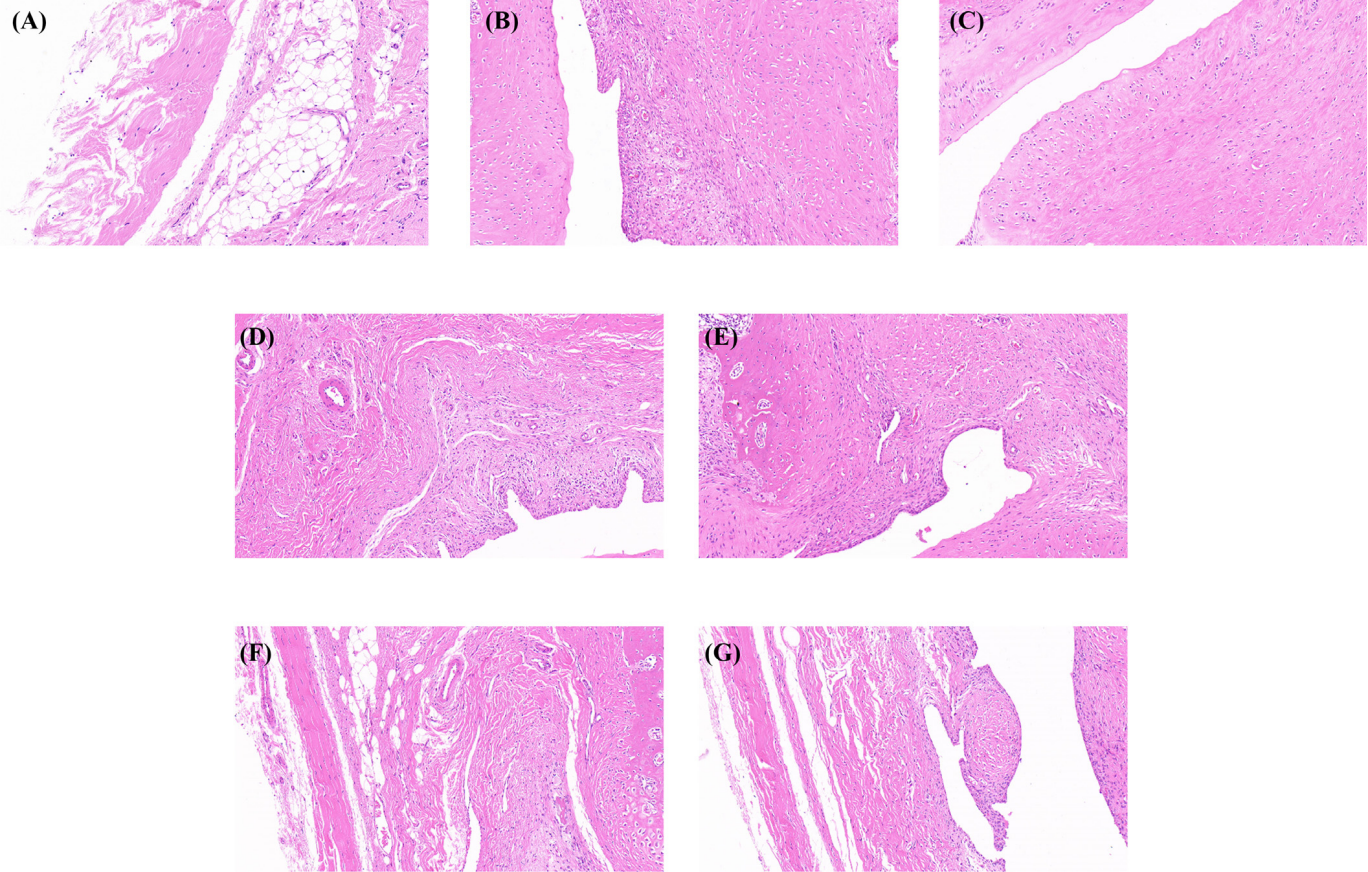
Effect of JLD on synovial tissues of rats with amelioration of gouty arthritis (HE, ×200): (**A**) Normal control group, (**B**) Model group, (**C**) JLD high-dose group, (**D**) JLD medium-dose group, (**E**) JLD low-dose group, (**F**) Etoricoxib group, (**G**) Pioglitazone group.

The normal control group showed a complete synovial epithelium arrangement, normal subsynovial vascular distribution, no obvious angiogenesis, and no inflammatory cell infiltration (Fig. **4**). In the model group, obvious pathological changes in arthritis were seen, the synovial epithelium was shed or even disappeared, and a large number of inflammatory cells (mainly neutrophils) were seen, accompanied by angiogenesis. Compared with the model group, the synovial epithelium of the etoricoxib group, pioglitazone group, and JLD group were relatively complete, synovial cell proliferation and inflammatory cell infiltration were reduced, and new small blood vessels were rarely seen. It was concluded that etoricoxib, pioglitazone, and JLD all improved local histopathological changes to a certain extent.

### 3.4 JLD can Reduce the Release of Inflammatory Factors

**Fig. (5):**
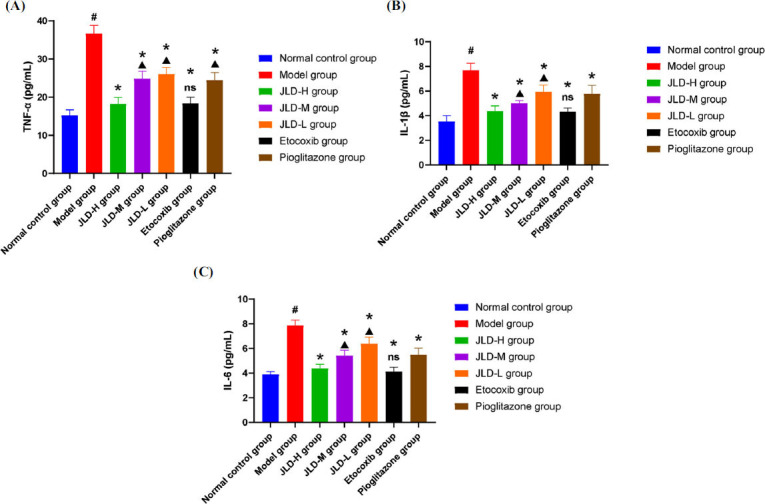
The levels of inflammatory cytokines in each group during *in vivo* experiment: (**A**) TNF-α; (**B**) IL-1β; (**C**) IL-6**;** (Compared with the normal control group, *^#^P<0.05*; Compared with the model group, *P<0.05; Compared with the high-dose group, *▲P<0.05*; Compared with the model group with no significant difference, ^ns^*P>0.05*).

**Fig. (6):**
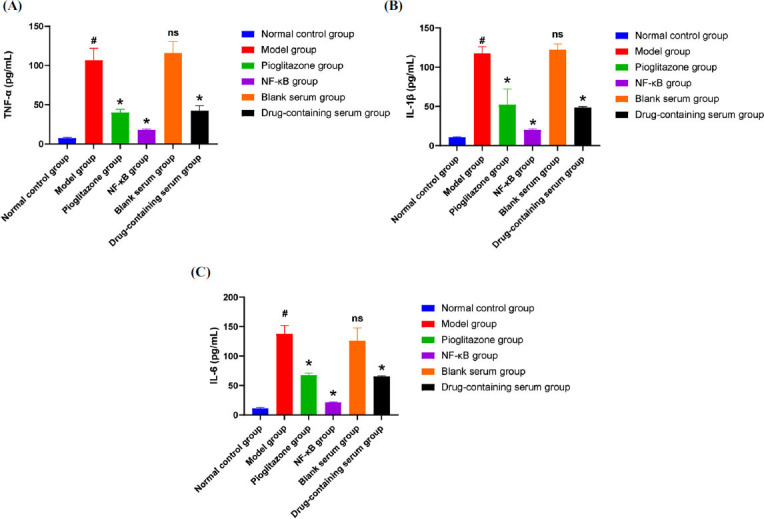
The levels of inflammatory cytokines in each group during *in vitro* experiment: (**A**) TNF-α; (**B**) IL-1β; (**C**) IL-6**;** (Compared with the normal control group, *^#^P<0.05*; Compared with the model group, *P<0.05; Compared with the model group with no significant difference, ^ns^*P>0.05*).

The release of inflammatory factors, such as TNF-α, IL-1β, and IL-6, were evaluated in both *in vivo* experiments and *in vitro* experiments. The results of *in vivo* experiments (Fig. **5**) showed that the levels of TNF-α (Fig. **5A**), IL-1β (Fig. **5B**), and IL-6 (Fig. **5C**) in the synovial tissue of rats in the model group were significantly increased compared with the normal control group, and the difference was statistically significant *(P<0.05)*. Compared with the model group, the levels of TNF-α, IL-1β, and IL-6 in the etoricoxib group, pioglitazone group, and the JLD groups were reduced, and the differences were statistically significant *(P<0.05)*. Compared with the JLD-H group, there was no significant difference in TNF-α, IL-1β and IL-6 in the etocoxib group *(P>0.05)*, and the levels of TNF-α, IL-1β, and IL-6 in the JLD-M and JLD-L groups were significantly increased *(P<0.05)*. The results of *in vitro* experiments (Fig. **6**) showed that compared with the normal control group, the levels of TNF-α (Fig. **6A**), IL-1β (Fig. **6B**), and IL-6 (Fig. **6C**) in macrophages in the model group were significantly increased, and the difference was statistically significant *(P<0.05)*, indicating that sodium urate can induce inflammation in J774.1 mononuclear macrophages. Compared with the model group, there was no significant difference in the inflammatory factors in the blank serum group *(P>0.05)*, indicating that rat serum did not significantly interfere with the test. Compared with the model group, the levels of TNF-α, IL-1β, and IL-6 in the NF-κB blocker group, pioglitazone group, and drug-containing serum group were significantly reduced, and the difference was significant *(P<0.05)*. From these findings, we speculate that the release of TNF-α, IL-1β, and IL-6 can be reduced *in vivo* and *in vitro*.

### 3.5 JLD can Up-regulate the Expression of PPARγ Protein and Down-regulate the Expression of LR4 and NF-κB Proteins

**Fig. (7):**
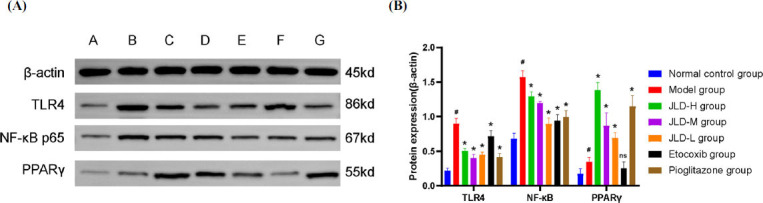
Effect of JLD on TLR4, NF-κB, and PPARγ protein *in vivo*. (**A**) TLR4, NF-κB, PPARγ, and β-actin protein expression in the synovium of rats in each group: A. Normal control group; B. Model group; C. JLD high-dose group; D. JLD medium-dose group; E. JLD low-dose group; F. Etoricoxib group; G. Pioglitazone group. (**B**) TLR4, NF-κB, and PPARγ protein expression levels (compared with the normal control group, *^#^P<0.05*; Compared with the model group, *P<0.05; Compared with the model group with no significant difference, ^ns^*P>0.05)*.

**Fig. (8):**
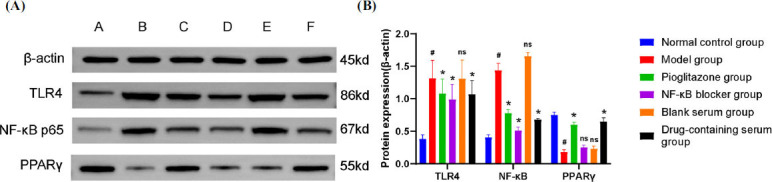
Effect of JLD on TLR4, NF-κB, and PPARγ protein *in vitro*. (**A**) TLR4, NF-κB, PPARγ, and β-actin protein expression in the synovium of rats in each group: A. Normal control group; B. Model group; C. Pioglitazone group; D. NF-κB blocker group; E. Blank serum group; F. Medicated serum group. (**B**) TLR4, NF-κB, and PPARγ protein expression levels (compared with the normal control group, *^#^P<0.05*; Compared with the model group, *P<0.05; Compared with the model group with no significant difference, ^ns^*P>0.05*).

In both *in vivo* and *in vitro* experiments, JLD can upregulate the expression of PPAR γ protein and downregulate the expression of TLR4 and NF-κB proteins. *In vivo* experimental results (Fig. **7A**, **B**) showed that compared with the normal control group, PPARγ protein expression in the model group was increased *(P<0.05)*, along with the TLR4 and NF-κB protein expressions *(P<0.05).* Compared with the model group, protein expression of PPARγ in JLD dose groups and pioglitazone group was significantly increased *(P<0.05)*, while protein expression of TLR4 and NF-κB was significantly decreased *(P<0.05)*. There was no up-regulation of PPARγ protein in the etoricoxib group. The *in vitro* experimental results (Fig. **8A**, **B**) demonstrated that compared with the normal control group, the expression of PPARγ protein in the model group was significantly reduced *(P<0.05)*, while the expression of TLR4 and NF-κB proteins was significantly increased (*P*<0.05). Compared with the model group, there were no differences in PPARγ, TLR4, and NF-κB proteins in the blank serum group, indicating that rat serum did not significantly interfere with the experiment.

The expression of PPARγ protein in the pioglitazone group and the drug-containing serum group was significantly increased compared with the model group *(P<0.05)*, and the expression of TLR4 and NF-κB protein was significantly decreased *(P<0.05)*. Moreover, the expression of PPARγ protein in the NF-κB blocker group was not significantly different *(P>0.05)*, while the expression of TLR4 and NF-κB proteins were decreased *(P<0.05)*. These results indicate that JLD prescription can activate the expression of PPARγ protein and inhibit the expression of TLR4 and NF-κB proteins in the treatment of acute gouty arthritis.

## 4 DISCUSSION

GA is an inflammatory joint disease caused by the abnormal metabolism of uric acid, leading to the deposition of urate salts in joints and other tissues [32, 33]. Its acute flares often manifest as redness, swelling, heat, and pain in the joints, most commonly affecting the first metatarsophalangeal joint, restricting mobility in the affected area, making walking difficult, and even causing fear of minor physical contact, severely impacting the patient's quality of life [34-37]. During the onset of gout, inflammation is the core pathological feature. Disruption in purine metabolism and/or reduced uric acid excretion leads to hyperuricemia [38, 39]. When hyperuricemia persists, monosodium urate crystals deposit around joints and can dislodge into the joint cavity upon certain stimuli. This activates various immune cells (such as macrophages, neutrophils, and synovial cells) that mediate inflammatory responses, recognizing and phagocytizing local monosodium urate deposits, thereby activating multiple intracellular inflammatory signaling pathways (such as Toll-like receptors, NLRP3 inflammasome), triggering GA [40-44]. Currently, clinical medications for GA include non-steroidal anti-inflammatory drugs (NSAIDs), corticosteroids, and colchicine, which rapidly alleviate the inflammation and pain of acute flares [45]. However, these drugs have significant side effects, including nausea, vomiting, gastrointestinal perforation, bone marrow suppression, and liver and kidney damage [8, 9]. Therefore, the current medical treatment of GA still has certain limitations.

Traditional Chinese medicine has a long history of treating gout. The characteristics of holistic treatment, a combination of disease and syndrome management with multiple biological effects, have contributed to its advantage of high efficacy and low toxicity in the prevention and treatment of gout, demonstrating the huge potential and development prospects of anti-GA therapies [46-50]. Simiao pills and Danggui Niantong decoction are commonly used in the treatment of gouty arthritis [31, 51]. Animal experiments have shown that Si Miao Tang can effectively reduce serum uric acid, liver XOD activity, foot thickness, serum IL-1β, and G-CSF levels in mice with gouty arthritis while also increasing pain thresholds and reducing serum TNF-α and IL-6 levels [52]. *In vitro* experiments have shown that Si Miao Pills significantly reduces the release of IL-1β in THP-1 cells inflamed by MSU crystals, suggesting a potential role in gout treatment [31]. This indicates that Si Miao Pills could potentially serve as anti-IL-1 agents for treating gout. A systematic review and meta-analysis indicated that Danggui Niang Tong Tang is effective in reducing blood uric acid and C-reactive protein levels in gout treatment and is considered safe [51]. Additionally, the Chinese herbal compound extract Tongfengkang (TFK) has been proven to alleviate joint swelling in rats with gouty arthritis and reduce levels of blood and urinary uric acid [53]. The levels of gout-related inflammatory factors were significantly lower than those in the control group, and further study of its mechanism of action revealed that TFK reduced the activation of macrophages and the aggregation of neutrophils in joint fluid, thereby exerting therapeutic effects.

Through literature research and clinical observation, our research group developed JLD based on the integration of Simiao San and Danggui Niantong Tang. Our research group has also carried out numerous experimental studies, and the research results have confirmed that JLD has good effects in the treatment of gouty arthritis, such as promoting blood circulation, reducing swelling, relieving pain, exerting anti-inflammatory effects, reducing blood uric acid, and inhibiting XOD activity. JLD aligns with the principles of traditional Chinese medicine. It is suitable for the pathogenesis of gouty arthritis (hyperuricemia, arthritis symptoms). Moreover, it has a relatively reliable clinical and experimental basis and is worthy of further research. However, the specific mechanism and target of its action remain unknown at present.

The experimental results demonstrated that in the rat model of acute gouty arthritis, JLD could reduce the ankle swelling index, prolong the painful tail-throwing time, and reduce blood uric acid levels. This is consistent with the previous experimental results of our group, suggesting that JLD reduces swelling, relieves pain, and lowers uric acid levels, with its effects strengthening progressively as the dose increases. After further pathological smear observation, it was found that JLD can also protect the integrity of synovial epithelium, reduce synovial cell and angiogenesis, decrease inflammatory cell infiltration, and improve the pathological changes of acute gouty arthritis. This effect is primarily analyzed through the regulation of inflammatory factors, such as TNF-α, IL-1β, and IL-6.

An increasing number of studies have reported that inflammatory response plays an important role in tissue damage induced by hyperuricemia [44, 54-56]. In patients with gouty arthritis, MSU crystals are formed and deposited in cartilage, the synovium, and around joints, and there is usually no inflammatory manifestation. Under the action of certain factors (such as cold, trauma, drinking, *etc*.), the crystals dislodge and enter the joint cavity, where they interact with macrophages, monocytes, neutrophils, and synovial cells. This interaction triggers the release of a variety of inflammatory mediators, such as histamine, prostaglandins, TNF-α, IL-1β, IL-6, *etc*., leading to an inflammatory cascade [57-59]. TNF-α has been shown to play an important role in acute inflammatory response of gout [60, 61]. IL-1β is considered to be the most classic inflammatory mediator of gouty arthritis. It initiates the inflammatory response and plays a key role in joint destruction, as well as in both acute and chronic gouty arthritis [62-64]. IL-6 is significantly expressed during the acute phase of gouty arthritis and is related to the activity of gouty arthritis [65-67]. The results of this experiment showed that JLD could reduce the levels of TNF-α, IL-1β, and IL-6 in both the rat model of acute gouty arthritis and the gout cell model established with MSU crystal-induced macrophages. Studies suggest that the pathogenesis and inflammatory manifestations of gouty arthritis may be closely related to its “TLR/NF-κB” signaling pathway.

TLRs recognize molecular indicators of cellular damage, such as MSU or protein components released by dead cells [68-71]. MSU may directly activate TLR or interact with certain cell surface proteins, such as binders, CD16, Fc receptor, or CD14 to activate TLRs, or other indirect pathways to activate TLRs and eventually activate NF-κB, mediating the gouty inflammatory response. Liu-Bryan R [72] reported that the expressions of TLR2, TLR4, and MyD88 play a key role in the inflammation induced by sodium urate crystals. Moreover, sodium urate crystals can act on chondrocytes through TLR2-MyD88 signal transduction, activating NF-κB and promoting the production of nitric oxide. This, in turn, facilitates chondrocyte calcification and lysis, contributing to the progression of gout into the chronic arthritis stage [73]. PPARγ in humans and rodents has anti-inflammatory properties. It can inhibit the inflammatory response by competitively inhibiting the expression of TLR4/NF-κB pathway [24, 74-76], and pioglitazone with PPARγ agonist effect can downregulate the expression of TNF-α and IFN-γ in synovial tissues of rat models of acute gouty arthritis [20]. Consequently, the TLR4/NF-κB signaling pathway and PPARγ were selected as the primary targets of this study. The results of this experiment showed that JLD could upregulate the expression of PPARγ protein in both the rat model of acute gouty arthritis and the gout cell model established by MSU crystal-induced macrophages, and its effect was similar to that of the PPARγ agonist pioglitazone. At the same time, it can also downregulate the expression of TLR4 and /NF-κB protein. In gout cell models, JLD can inhibit the nuclear translocation of NF-κB, and its effect is similar to that of NF-κB blocker SN50. Based on these findings, we speculate that the mechanism of the treatment of acute gouty arthritis may partly lie in upregulating the expression of PPARγ and inhibiting the expression of the TLR4/NF-κB signaling pathway. Notably, it is possible that the agonist effect of JLD may be attributed to its binding affinity to the target rather than being solely concentration-dependent. This hypothesis could be further investigated through receptor binding assays by comparing concentration-response data with binding affinity data. We plan to address this aspect in future studies.

## CONCLUSION

In summary, we conclude that JLD demonstrates significant potential in the treatment of acute gouty arthritis by modulating key inflammatory pathways. Our study reveals that JLD alleviates acute gouty arthritis symptoms by promoting the expression of PPARγ, which, in turn, inhibits the TLR4/NF-κB signaling pathway. This molecular mechanism results in reduced inflammation, lowered serum uric acid levels, and decreased joint swelling. These findings provide valuable insight into the therapeutic effects of JLD in managing gout and suggest that it may serve as a promising adjunct to current treatment strategies. However, further studies are needed to fully elucidate its long-term effects and the precise molecular targets involved, paving the way for future clinical applications.

## STUDY LIMITATIONS

This study demonstrates the efficacy of JLD in alleviating AGA through PPARγ-mediated TLR4/NF-κB inhibition; however, several limitations exist, such as model constraints: rat models may not fully replicate human gout pathophysiology (*e.g*., uricase deficiency in humans); mechanistic specificity: unidentified bioactive compounds in JLD responsible for PPARγ activation warrant phytochemical isolation, and clinical translatability: Short-term interventions lack long-term toxicity and efficacy data for chronic gout. Further human trials, compound screening, and investigations into the crosstalk between PPARγ and NLRP3 are needed.

## Figures and Tables

**Table 1:** Sources of the reagents.

**Reagent Name**	**Source/Supplier**	**Batch/Catalog No.**
Potassium oxonate	Shanghai Hanbo Trading Co., Ltd	131015
Monosodium urate	Beijing Sailaibao Technology Co., Ltd	U8290
Pioglitazone hydrochloride	Changsha Keno Biotechnology Co., Ltd	A0908AS
TNF-α ELISA Kit	Andy Biotechnology (Shanghai) Co., Ltd	DZE20220
IL-1β ELISA Kit	Andy Biotechnology (Shanghai) Co., Ltd	DZE20533
IL-6 ELISA Kit	Andy Biotechnology (Shanghai) Co., Ltd	DZE20D1
PPARγ antibody	Abcam, USA	ab209350
TLR4 antibody	Abcam, USA	ab13867
NF-κB p65 antibody	Abcam, USA	ab31482
β-actin antibody	Abcam, USA	ab209729
Fetal bovine serum	Changsha Keno Biotechnology Co., Ltd	10099-141
DMEM medium	Changsha Keno Biotechnology Co., Ltd	10569-044
Penicillin-Streptomycin	Changsha Keno Biotechnology Co., Ltd	1805240107
SN50(NF-κB inhibitor)	Shanghai Unibio Biotechnology Co., Ltd.	135854

**Table 2:** The composition of JLD.

**Chinese Name**	**Latin Name**	**Medicinal Part**	**Daily Adult Dose (g)**	**Picture**
Cangzhu	*Atractylodes lancea* (Thunb.) DC.	Dried rhizomes	20	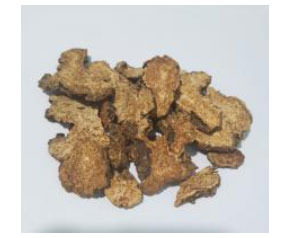
Huangbo	*Phellodendron chinense* Schneid.	Dried bark	10	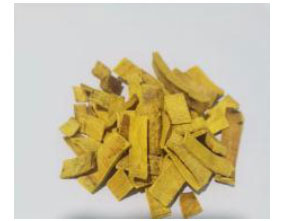
Huangqin	*Scutellaria baicalensis* Georgi	Dried root	10	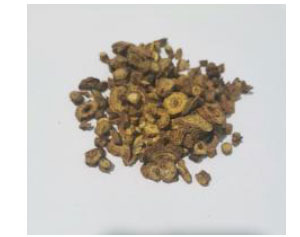
Tufuling	*Smilax glabra* roxb.	Dried rhizomes	15	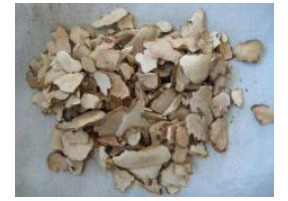
Yinchen	*Artemisia capillaris* Thunb.	Dry aboveground parts	15	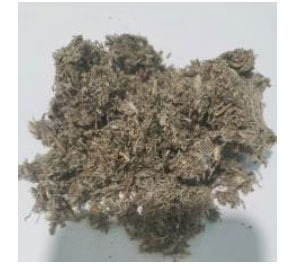
Fangji	*Stephania tetrandra* S. Moore	Dried root	10	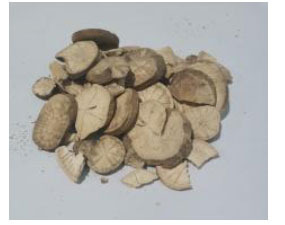
Zexie	*Alisma orientalis*(Sam.)Juzep.	Dried tuber	10	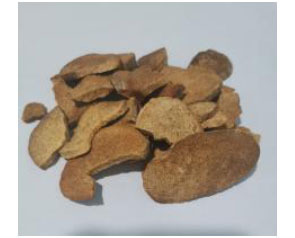
Baizhu	*Atractylodes macrocephala* Koidz.	Dried rhizomes	15	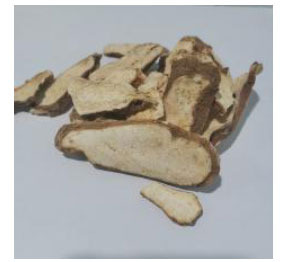
Danggui	*Angelica sinensis* (Oliv.) Diels.	Dried rhizomes	6	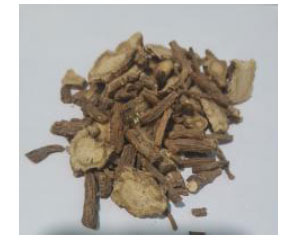
Gancao	*Glycyrrhiza uralensis* Fisch.	Dried roots and rhizomes	6	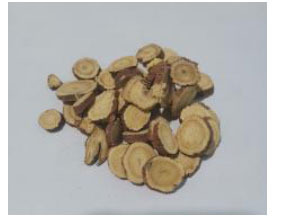

## Data Availability

All data generated or analyzed during this study are included in this published article.

## LIST OF ABBREVIATIONS

AGA: Acute gouty arthritis
JLD: Juanbi Lijieqing Decoction
MSU: Monosodium urate
NF-κB: Nuclear factor kappa B
PPARγ: Peroxisome proliferator-activated receptor gamma
TLR4: Toll-like receptor 4
